# Reciprocal effects between loneliness and sleep disturbances from adolescence to mid-adulthood: the HUNT study

**DOI:** 10.1093/sleepadvances/zpag004

**Published:** 2026-01-12

**Authors:** Nayan Parlikar, Joanna McHugh Power, Philip Hyland, Andrew N Coogan, Kirsti Kvaløy, Linn Beate Strand, Geir Arild Espnes, Steinar Krokstad, Unni Karin Moksnes

**Affiliations:** Department of Public Health and Nursing, Faculty of Medicine and Health Sciences, Norwegian University of Science and Technology, Trondheim, Norway; Department of Psychology, Maynooth University, Maynooth, Kildare, Ireland; Department of Psychology, Maynooth University, Maynooth, Kildare, Ireland; Department of Psychology, Maynooth University, Maynooth, Kildare, Ireland; Department of Public Health and Nursing, Faculty of Medicine and Health Sciences, Norwegian University of Science and Technology, Trondheim, Norway; HUNT Research Centre, Department of Public Health and Nursing, Norwegian University of Science and Technology, Levanger, Norway; Levanger Hospital, Clinic for Mental Health and Substance Use, Nord-Trøndelag Hospital Trust, Levanger, Norway; Department of Public Health and Nursing, Faculty of Medicine and Health Sciences, Norwegian University of Science and Technology, Trondheim, Norway; Department of Public Health and Nursing, Faculty of Medicine and Health Sciences, Norwegian University of Science and Technology, Trondheim, Norway; Department of Public Health and Nursing, Faculty of Medicine and Health Sciences, Norwegian University of Science and Technology, Trondheim, Norway; HUNT Research Centre, Department of Public Health and Nursing, Norwegian University of Science and Technology, Levanger, Norway; Levanger Hospital, Clinic for Mental Health and Substance Use, Nord-Trøndelag Hospital Trust, Levanger, Norway; Clinic for Mental Health and Substance Use, Nord-Trøndelag Hospital Trust, Levanger, Norway; Department of Public Health and Nursing, Faculty of Medicine and Health Sciences, Norwegian University of Science and Technology, Trondheim, Norway

**Keywords:** loneliness, sleep disturbances, longitudinal, reciprocal, adolescence, adulthood, mediation, anxiety, depression

## Abstract

**Study Objectives:**

Loneliness and sleep disturbances are prevalent and interrelated public health concerns, especially during the transition from adolescence to adulthood, a period marked by major psychological and social changes. However, less is known about their potential reciprocal relationship across development. This study examines the longitudinal, bidirectional associations between loneliness and sleep from adolescence to mid-adulthood using data from the Trøndelag Health Study (HUNT).

**Methods:**

We used data from Young-HUNT1 (1995–1997), HUNT3 (2006–2008), and HUNT4 (2017–2019), including 2185 participants (60.6% female). Loneliness and sleep were measured using harmonized items across waves. Structural equation modeling tested cross-lagged associations between loneliness and sleep, adjusting for sociodemographic and mental health covariates. Mediation analyses explored the underlying mechanisms of anxiety and depression.

**Results:**

Loneliness declined over time, while sleep showed a fluctuating pattern. Both constructs demonstrated high temporal stability. Loneliness did not significantly predict future sleep problems; however, sleep in early adulthood was a predictor of increased loneliness in midlife. Cross-sectional associations between loneliness and sleep were significant in adolescence and mid-adulthood. Mediation analyses showed that anxiety and depression mediated the longitudinal links between loneliness and sleep disturbances across different life stages.

**Conclusions:**

Loneliness and sleep are stable and interrelated across development. Sleep problems in early adulthood appear to be a stronger predictor of future loneliness, highlighting sleep as a potential intervention target to reduce long-term social and mental health difficulties. Symptoms of anxiety and depression mediated these associations, indicating key psychological pathways linking loneliness and sleep over time.

Statement of Significance Loneliness and sleep disturbances are prevalent and interrelated public health concerns, especially during the transition from adolescence to adulthood, a period marked by intense psychological and social change. Despite growing recognition of their individual effects, little is known about how loneliness and sleep interact longitudinally across development. This study is among the first to investigate their reciprocal associations from adolescence to mid-adulthood, providing a novel developmental perspective that bridges gaps between mental health, sleep research, and social functioning. Future work should build on these findings to identify sensitive windows for prevention and clarify the mechanisms through which early sleep patterns influence long-term social and emotional outcomes.

## Introduction

The transition from adolescence to adulthood involves major physical, social, emotional, and psychological changes, often challenging individuals’ capacity for social connection and emotional well-being [1]. Among these, loneliness is a common and distressing experience with lasting effects on health and well-being [2, 3]. Loneliness is typically defined as a qualitative, subjective discrepancy between individuals’ expectations and satisfaction with actual social relationships [4, 5] and is often heightened in adolescence due to developmental changes and shifting social dynamics [6–8]. Adolescence, typically defined as ages from 10-24 years [9], is marked by greater reliance on peers and romantic relationships and reduced closeness with parents [10]. As individuals move into early adulthood (18–35 years) and mid-adulthood (36–55 years) [11], new roles related to education, work, and family reshape social networks [12, 13]. Despite increased autonomy and opportunities for connection, maintaining meaningful relationships often becomes difficult which may lead to isolation, yielding experiences of loneliness [6]. Social isolation and loneliness are distinct constructs, and objective isolation, such as reduced social contact, has been associated with increased subjective isolation in early adulthood [14]. While loneliness is widely recognized as a public health concern, most research has focused on its unidirectional links to later health outcomes, often within limited age ranges and short follow-up periods [15–17]. Moreover, the reciprocal association between loneliness and health issues across critical development periods remains insufficiently understood.

Loneliness is linked to adverse health outcomes through mechanisms such as poor immune function, inflammation, stress, and sleep disruption [18]. On the other hand, health issues can also contribute to loneliness through restricted social participation and reduced sense of belonging [19–21]. In this context, sleep disturbances are particularly interesting because they are both a potential consequence and predictor of loneliness [22]. During adolescence and early adulthood, biological changes in sleep architecture and psychosocial factors, such as academic stress, irregular routines, social media use, and poor mental health can disrupt sleep [23–27]. Given the strong links between sleep and both physical and mental health outcomes [28–30], understanding the developmental and psychological factors that underlie sleep problems is vital. Moreover, studying sleep and loneliness across multiple life stages, rather than at a single time point, provides crucial insight into how vulnerabilities early in life contribute to later life outcomes. Life course approaches also capture transitions (e.g. workforce entry, parenthood, caregiving) that uniquely affect sleep and social connectedness [24, 31]. Without this long-term perspective, we may overlook cumulative risks or miss optimal timing for interventions.

Loneliness and sleep disturbances are linked across the lifespan, with loneliness predicting greater difficulty initiating and maintaining sleep, as well as nonrestorative sleep [32–34]. From an evolutionary perspective, it is theorized that humans have relied on a safe environment to survive and thrive [35–37]. The absence of secure social surroundings may result in feelings of loneliness, which in turn, heighten feelings of vulnerability and unconscious vigilance to social threats, impairing sound sleep [37]. Stress and inflammatory responses via the hypothalamic–pituitary–adrenal (HPA) axis and cortisol release may partly explain this association [35, 38, 39], thereby depleting individuals physically and contributing to daytime exhaustion [37]. This in turn affects sleep quality and causes additional worries of difficulty falling asleep [40]. Furthermore, from a psychophysiological perspective, loneliness can lower immune system function and raise neuroendocrine dysregulation [41], both of which are risk factors for sleep problems. Emerging evidence also suggests that poor sleep can predict increased loneliness at all life stages. Sleep disturbances, particularly when persistent, have been shown to reduce empathy, increase interpersonal sensitivity [42], and negatively impact brain regions like the amygdala and prefrontal cortex, leading to emotional dysregulation [43] which may hinder social interaction and lead to social withdrawal [44, 45], gradually contributing to increased loneliness. In addition to the direct associations, longitudinal studies show that both loneliness and sleep disturbances share common pathways through psychological distress, including depression, anxiety, heightened rumination, reduced resilience and chronic stress [46–48]. Loneliness is associated with symptoms of anxiety and depression, which can heighten physiological arousal and cognitive rumination, thereby disrupting sleep [47, 49]. Conversely, sleep disturbances may exacerbate emotional dysregulation and negative behaviour, reinforcing feelings of social withdrawal and loneliness [50].

Although previous studies have demonstrated associations between loneliness and sleep disturbances across different populations, the direction and timing of this relationship remain unclear. Some research suggests that loneliness is associated with subsequent sleep problems [19, 22, 51–54], but other studies have not found this link [55]. Similarly, sleep disturbances may be associated with subsequent loneliness and issues with belongingness, but the evidence for the direction of pathway remains scarce [19, 22, 56–58]. Given this complexity, research should move beyond one-way models and explore the long-term interplay and bidirectionality of loneliness and sleep over time to reveal key pathways that shape long-term health outcomes. Building on these gaps, the present study aimed to examine the temporal ordering and reciprocal relationships between loneliness and subjective sleep disturbances in the Norwegian population from adolescence to adulthood using a cross-lagged panel model. Based on the literature we describe above, we hypothesized that (a) higher levels of loneliness would be associated with greater subjective sleep disturbances both cross-sectionally and longitudinally from adolescence to adulthood; (b) both loneliness and sleep disturbances would show temporal stability, with each predicting itself over time, and (c) loneliness would be a stronger predictor of subsequent sleep disturbances than the reverse, based on the model developed by Cacioppo and Hawkley (2003) [35, 59].

## Methods

### Study design and participants

This study takes data from the Trøndelag Health Study (HUNT), a large, population-based longitudinal survey conducted in Trøndelag County, Norway. The HUNT Study consists of four health surveys carried out at 11-year intervals, with Young-HUNT targeting adolescents (13–19 years) and HUNT for adults (20 + years) from Trøndelag County in Norway. The Young-HUNT Study comprises three different waves: Young-HUNT1 (1995-97), Young-HUNT3 (2006-08), and Young-HUNT4 (2017-19). We used data from three waves: Young-HUNT1 (YH1; 1995–1997), HUNT3 (H3; 2006–2008), and HUNT4 (H4; 2017–2019). These waves enable a follow-up from adolescence into mid-adulthood. Participants from YH1 were followed longitudinally into H3 and H4, allowing analysis of how adolescent loneliness relates to sleep disturbances later in life. In each wave, participants completed two questionnaires (Q1 and Q2). Q1 was distributed to all participants and covered a broad range of general health topics, including demographic information, lifestyle factors, self-rated health, and common mental and physical symptoms. Q2, a follow-up questionnaire sent to those who responded to Q1, gathered more detailed information on specific health domains. Both Q1 and Q2 included key questions relevant to this study, such as those assessing loneliness (e.g. “Do you often feel lonely?”) and sleep disturbances (e.g. problems falling asleep, waking up too early, or feeling tired during the day). Details on the HUNT and Young-HUNT Study (including the cohort profile, recruitment procedures, eligibility criteria, response rates, non-response bias, reasons for data collection and methods, variables measured, areas of research, key findings, strengths and weaknesses) are given elsewhere [60–62]. A total of 8980 participants completed the survey at YH1. From the YH1 cohort, 1919 individuals participated in H3 (mean baseline age: 15.9), and 1906 completed both Q1 and Q2 at both time points. The final analytic sample consisted of 2185 participants who completed assessments at all three time points, allowing for a comprehensive examination of associations between loneliness and sleep from adolescence to adulthood.

*Non-Participants at H3:* H3 survey (2006–08) was conducted in Nord-Trøndelag, with ~54% of all invited adults participating, thus necessitating a small non-participant follow-up conducted to assess characteristics of non-responders. Given the overall lower response rate in H3 survey, the response among those aged 20–29 years in H3 was also low (31.5%), largely due to out-migration for education [61]. About nine months after the H3 survey, a non-participant questionnaire (QNP) was mailed to all eligible non-responders at H3, including core items from Q1 and Q2 on symptoms, diseases, lifestyle, and reasons for nonparticipation. The QNP yielded an additional 1439 responses, increasing the YH1–H3 follow-up to 3358 participants. Most of the participants in this survey did not take the main HUNT3 survey due to reasons like they did not get the invitation, or they did not have the time. A few of them did not believe in participating in surveys and had sick children. A few others thought that they did not benefit from such surveys and had other miscellaneous reasons [63]. Compared with participants, non-participants were generally more likely to report poorer self-rated health, higher prevalence of chronic diseases, greater mental distress, and more frequent insomnia complaints. They also tended to have lower socioeconomic status (SES), lower levels of physical activity, and higher rates of smoking and alcohol use. These differences align with previous reports showing that participation rates in HUNT3 were lowest among younger adults (31.5% in the 20–29-year group) and that non-participants were overall in poorer health than participants [63]. Further details on non-participants and group differences are reported elsewhere [63].

The Emigrant study and Sør-Trøndelag study at H4: During H4, in addition to the local residing population, individuals who had moved out of Trøndelag were invited to the HUNT4 Emigrants Study and HUNT4 Sør-Trøndelag which covered the participants in Trondheim in 2019 to examine potential differences in health, behaviour, and socioeconomic conditions between those who had emigrated and those still residing in Nord-Trøndelag. All former residents who had previously participated in any of the earlier HUNT surveys (HUNT1–3 or YoungHUNT1–3) were invited to participate via mail [60].

**Figure 1:**
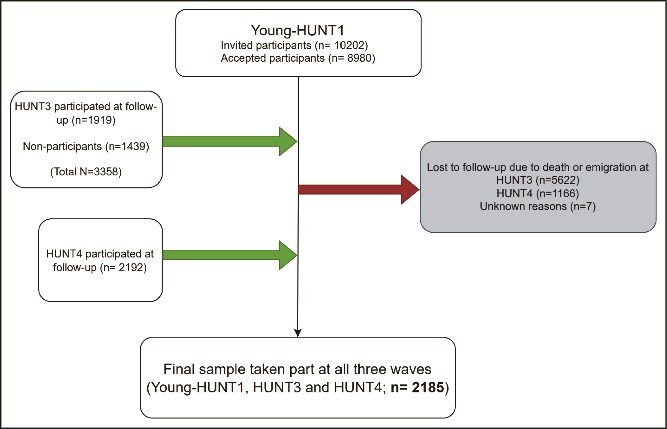
Flowchart of participant inclusion across the three waves of the HUNT study.

Participation in the HUNT Study was voluntary, and all participants signed a written consent form. Written consent from parents or a guardian was required for participants under the age of 16 years. The Regional Committee for Medical and Health Research Ethics approved the present study. The flowchart of participation shows the progression of participants from YH1 to H4, highlighting the response rates at each stage and the inclusion of non-responders (Figure 1).

### Measures

**Loneliness:** Loneliness was assessed using a single-item measure, *“Do you feel lonely?”*. At YH1, the loneliness measure had five response categories [1]*: Very rarely or never* [2], *Rarely* [3], *Sometimes* [4], *Often, and* [5] *Very often*. To ensure consistency with loneliness measures at H3 and H4, which used a four-point Likert scale (*1 = No Loneliness*, *2 = A little 3 = A good amount and 4 = Very much*), responses at YH1 were re-coded by grouping *“Very rarely or never”* and *“Rarely”* as *“No loneliness.”* Loneliness was treated as a manifest variable in analyses. A manifest variable is an observable variable, i.e. a variable that can be measured directly. A manifest variable can be continuous or categorical [64].

**Sleep Disturbances:** These were assessed through two self-reported items at all three time points: *“Have you had difficulty falling asleep?”* and *“Have you woken too early and not been able to fall asleep again?”*. At YH1, responses were recorded based on sleep difficulties experienced in the past month using four categories [1]*: Almost every night* [2], *Often* [3], *Sometimes, and* [4] *Never*. To align with the three-category response scale used at H3 and H4 (*1 = Never, 2 = Sometimes, 3 = Often), “Almost every night”* and *“Often”* were combined at YH1. At H3 and H4, sleep disturbances were assessed based on difficulties experienced in the past three months. A mean score (range 1–6) was calculated from the summed items at each time point, consistent with prior literature [65, 66]. To examine the prevalence of sleep disturbances, participants were classified as having sleep disturbances if they answered *“Often,” “Almost every night”, “Several times a week”, or “At least 3 times a week”* to at least one of the questions on at least one item, even with one missing response. Conversely, if a participant answered *“Never,” “Rarely,”* or *“Sometimes”* to one question and did not respond to the other, they were not classified as having sleep disturbances. This approach is consistent with past uses of this scale in the literature [67]. Sleep disturbances were modeled as a latent variable, allowing correlated residuals across time to account for shared measurement error. A latent variable is a variable that is not directly observed, but is inferred from other variables that can be directly measured [64].

**Demographics:** Sociodemographic characteristics, including sex, age, living status, educational plans as a proxy for SES were adjusted for during the period from YH1 to H3; sex and age were adjusted for from H3 to H4.

SES was measured by adolescents’ future education plans at YH1. The variable included five categories [1]*: None* [2], *College or university less than 4 years* [3], *College or university for 4 years or more* [4], *Vocational school or training and* [5] *Don’t know*. College or university education of fewer than 4 years and for 4 years or more was “College or higher education”, which was a proxy for high SES. Rest of the categories were classified as “Secondary school or less or vocational school”. Education plans or expectation of education is frequently used as an indicator of SES in epidemiological studies [68–71].

**Symptoms of anxiety and depression:** At YH1, mental distress in the form of anxiety and depression was measured by using the 5-item Hopkins Symptom Checklist (HSCL-5). The Hopkins Symptom Checklist-25 (HSCL-25) is an extensively used self-report measure of anxiety and depression symptoms. Compared with the HSCL-25, the short-form model fit is good with acceptable validity [72]. The adolescents were asked if they had experienced each of the following during the last 14 days: *“Been constantly afraid and anxious”, “Felt tense or uneasy”, “Felt hopeless about the future”, “Felt dejected or sad”, “Worried too much about various things”*. Each item was answered on a four-point scale [1]*: Not at all* [2], *A little* [3], *Quite a bit, and* [4] *Very much* with the cut-off score ≥ 2.

At H3, anxiety and depression were measured by single questions from the Q1 by the CONOR-Mental Health Index (CON-MHI), Q2 by the HSCL, and CON-MHI among non-participants. The questions were: *“Have you felt anxious in the last two weeks?”* (CON-MHI), *“Have you been constantly afraid and anxious in the last 14 days?”* (HSCL-5), *“Have you felt down and depressed in the last two weeks?”* (CON-MHI) *and “Have you felt down and sad in the last 14 days?”* (SCL-5). Responses were recorded on a scale from (*1 = No* to *4 = Very much*) on all scales.

Full coding for all covariates is provided in Table 1.

**Table 1:** Characteristics of the study sample that commonly participated at all three time points

Variables (n = 2185)	Young-HUNT1	HUNT3	HUNT4
Age (Mean, SD)	16.25 (1.82)	27.27 (1.92)	37.97 (1.98)
Sex (% females)	60.6%		
Loneliness			
Mean score, SD	1.35 (0.62)	1.26 (0.54)	1.23 (0.53)
N (%)			
*No*	1549 (70.9%)	1690 (77.3%)	1739 (79.6%)
*A little*	499 (22.8%)	393 (18%)	335 (15.3%)
*A good amount*	87 (4%)	70 (3.2%)	55 (2.5%)
*Very much*	29 (1.3%)	12 (0.5%)	18 (0.8%)
*Missing (n, %)*	21 (1%)	20 (0.9%)	38 (1.7%)
Sleep disturbances			
Mean score	2.97 (0.98)	2.74 (0.93)	2.87 (1.01)
(n,%)^*^	237 (10.8%)	160 (7.3)	265 (12.1)
*Missing (n, %)*	15 (0.7%)	381 (17.4%)	22 (1%)
Mental distress	1.46 (0.48)^*^^*^		
Mean (SD) HSCL≥2 (n,%)	331 (15.1)		
*Missing (n, %)*	49 (2.2)		
Anxiety			
(Mean, SD)		1.16 (0.46)^*^^*^^*^	1.17 (0.47)^*^^*^^*^^*^
*Missing (n, %)*		7 (0.3%)	32 (1.5)
Depression			
(Mean, SD)		1.42 (0.65)^*^^*^^*^	1.34 (0.58)^*^^*^^*^^*^
*Missing (n, %)*		6 (0.3)	32 (1.5)
Plans for education (n,%)			
*Secondary school or less or vocational school*	1453 (66.5)		
*College or higher education*	661 (30,2)		
*Missing*	71 (3.2)		
Number of friends			
*None*	25 (1.1)		
*One*	108 (4.9)		
*Several*	2020 (92.4)		
*Missing*	32 (1.5)		
Sibling relationships			
*No siblings*	66 (3)		
*Bad*	83 (3.8)		
*Average*	1181 (54.1)		
*Better than usual*	816 (37.4)		
Living alone	104 (4.8)		

^*^Participants were classified as having sleep disturbances who responded “Often” or “Almost every night” or “Several times in a week” or “At least 3 times a week” to at least one of the questions. If missing on one question, then if they answered “Often” or “Almost every night” or “Several times in a week” or “At least 3 times a week” to one of the questions and did not answer the other, they were classified as having sleep disturbances . If they answered “Never” or “Rarely” or “Sometimes” to one of the questions and did not answer the other, they were not included in sleep disturbances .

^**^Young-HUNT1: Anxiety and depression measured by HSCL-5. ^***^HUNT3: Anxiety and depression measured by HUNT3 Q1(CON-MHI), Q2 (HSCL) and HUNT3 non-participants Q1 (CON-MHI). ^****^HUNT 4: Anxiety and depression measured by HUNT4 Q1, HUNT4 Emigrants Q1 and HUNT4-ST Q1 (CON-MHI).

SD: Standard deviation

### Statistical analysis

All analyses were conducted using R version 3.6.3 (R Foundation for Statistical Computing, Vienna, Austria). We employed structural equation modeling (SEM) with robust maximum likelihood estimation (MLR) estimator using the lavaan package [73]. A cross-lagged panel modeling (CLPM) framework was used to examine the longitudinal and reciprocal associations between loneliness and sleep disturbances across three time points [74]. The model allowed for the examination of the association between loneliness and sleep disturbances at each time-point, the association of each variable with itself across time (autoregressive pathways), and their longitudinal prediction of one another (cross-lagged pathways). Loneliness, anxiety, depression, and sleep disturbances were measured concurrently at each wave (YH1, H3, H4). Thus, the temporal ordering of these constructs within-wave cannot be empirically determined. We therefore conceptualized anxiety and depression as integral components of the loneliness–sleep framework, reflecting both their potential confounding and mediating roles. This dual modeling approach acknowledges the strong psychological interconnections among these constructs and allows both direct and indirect pathways to be evaluated within the same theoretical framework.

Initially, we specified an autoregressive model to examine the stability of loneliness and sleep disturbances over time. This model included both unadjusted and adjusted models for sex, age, educational plans, living status, anxiety, and depression. Specifically, anxiety and depression were included as confounders in longitudinal paths to account for concurrent psychological symptoms that may influence both loneliness and sleep across waves. We then extended the model by adding cross-lagged paths to assess the potential bidirectional influences between loneliness and sleep disturbances over time. Fit indices for both models were compared to determine whether the inclusion of cross-lagged effects improved model fit. Nonetheless, adjusted cross-lagged effects were examined separately for exploratory and theoretical interest, given prior research suggesting reciprocal relationships between the two constructs. Additionally, cross-sectional associations between loneliness and sleep disturbances were examined at each time point to provide a comprehensive understanding of their relationship at different stages. These cross-sectional analyses were conducted using multivariable regression analyses, adjusting for relevant covariates. Model fit was assessed using multiple indices: Chi-squared test (χ^2^), Comparative Fit Index (CFI) and Tucker-Lewis Index (TLI), where values ≥0.90 indicate acceptable fit; Root Mean Square Error of Approximation (RMSEA) and Standardized Root Mean Square Residual (SRMR), where values ≤0.08 suggest good fit; and the chi-square test, where a non-significant value suggests adequate fit [75–78]. Additionally, the Akaike Information Criterion (AIC) and Bayesian Information Criterion (BIC) were used to compare models, with lower values indicating a better balance between model fit and complexity.

*Mediation analyses:* In the mediation analyses, we explicitly modeled anxiety and depression as mediators to test the indirect pathways linking loneliness and sleep disturbances across waves, given theoretical frameworks that position mental health as a possible pathway through which loneliness may influence sleep [79–81], This approach allows us to reflect the central theoretical role of anxiety and depression while acknowledging temporal ambiguity and allowed us to assess the plausibility of an indirect effect, without assuming these variables were purely confounding in nature.

To address the effect of other confounders which are similar to loneliness, like the social-context variables (number of friends and sibling relationship quality), we also conducted sensitivity analyses including the social-context variables.

Sibling relationships were measured by the question, *“If you have siblings, how good a relationship do you feel you have with your sister or brother?”* with response options [1] *Much worse than normal* [2] *Worse than normal* [3] *Average* [4] *I do not have siblings* [5] *Better than normal* [6] *Much better than normal*. Categories [1] and [2] were classified as “Bad” and categories [5] and [6] as “Better than usual”. Participants were also asked to report on the number of friends they had. (Analyses not shown).

## Results

### Demographics of study sample and prevalence of loneliness and sleep disturbances

The study tracked changes in loneliness and sleep disturbances over several years. Loneliness generally declined, while sleep patterns were more varied. Participation dropped over time, especially among younger adults who moved away or people facing health or lifestyle challenges (Table 1). The participation rate in H3 at follow-up from YH1 was low (n = 1919 at H3 and n = 1439 at the H3 Non-Participants study), given the overall low participation rate in H3 especially among 20–29-year-olds (31.5%) [63]. Many people in this age group had moved out of the county for further education and were not eligible for invitation to the H3 survey [61]. Attrition from H3 to H4 was moderately high among people with chronic diseases or poor self-rated health or who were smokers [60]. Further attrition analyses showed that attrition over two decades was more pronounced among individuals with early indicators of psychological distress, poor social relationships, and unhealthy lifestyle factors. Between YH1 and H3, participants who dropped out were more likely to be male, from lower socioeconomic backgrounds, not living with parents or family, and from divorced households. They also reported lower life satisfaction, fewer close friendships, poorer self-rated health, limited outdoor activity, and lower participation in organized social groups. Between H3 and H4, dropout was associated with financial strain, workplace problems, high BMI, and high anxiety and depression symptoms. This selective loss likely biases the analytic sample toward individuals with greater psychological resilience and more stable life circumstances, leading to conservative estimates of the associations examined (Supplementary file; Tables S1 and S2).

### Model fit

The measurement model for sleep disturbances as a latent variable showed good fit, with moderate, significant factor loadings (0.546–0.715). The baseline autoregressive model with stability paths for loneliness and sleep also fit well. Adding cross-lagged paths from sleep to later loneliness resulted in a minor decline in fit, while paths from loneliness to later sleep disturbances led to a more noticeable deterioration. We tested cross-lagged associations in separate models, excluding stability paths. The model testing whether loneliness predicted later sleep showed good fit and the reverse model, i.e. sleep disturbances predicting later loneliness, demonstrated excellent fit. (Fit indices for measurement, autoregressive, and cross-lagged structural equation models examining associations between loneliness and sleep disturbances across three waves are attached in the Supplementary material Table S3).

### Autoregressive pathways

**Figure 2:**
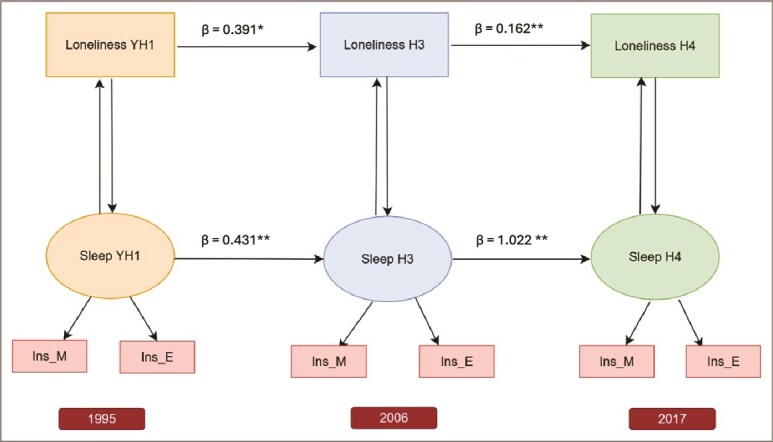
Adjusted autoregressive and cross-sectional associations between loneliness and sleep disturbances across three time points in the HUNT study (YH1: Young-HUNT1 in 1995, H3: HUNT3 in 2006, H4: HUNT4 in 2017). Sleep disturbances are indicated by two items: Ins_M (“Had difficulty falling asleep?”) and Ins_E (“Woke too early and couldn’t get back to sleep?”). Cross-sectional associations at each wave and autoregressive paths across waves are presented. Cross-sectional association between loneliness and sleep disturbances at YH1 is adjusted for sex, age, educational plans, living alone, and mental distress in adolescence. Cross-sectional association at H3 is adjusted for sex, age, anxiety, and depression. Cross-sectional association in H4 is adjusted for sex, age, education, income, depression, and anxiety. All autoregressive pathways are adjusted for the same covariates as the corresponding wave from which the path originates.^*^p < .05, ^**^p < .001. Arrows without asterisks represent non-significant paths.

The model showed significant temporal stability in both loneliness and sleep disturbances. Loneliness in adolescence (YH1) moderately predicted loneliness in early adulthood (H3) (β = 0.391, *p*=.006), and loneliness at H3 predicted loneliness in later adulthood (H4), though with a smaller effect (β = 0.162, *p*<.001). Sleep disturbances also showed strong continuity: adolescent sleep disturbances predicted early adulthood sleep disturbances (β = 0.431, *p*<.001), and the effect from early to mid-adulthood was particularly strong (β = 1.022, *p*<.001) (Figure 2).

### Cross-lagged panel models

**Figure 3:**
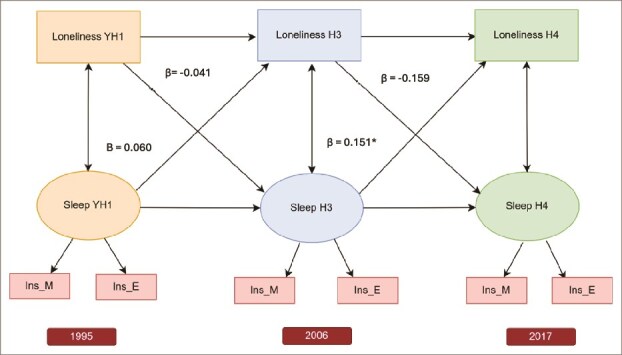
Adjusted cross-lagged panel model examining directional associations between loneliness and sleep disturbances across three time points in the HUNT study (YH1: Young-HUNT1 in 1995, H3: HUNT3 in 2006, H4: HUNT4 in 2017). Sleep disturbances are indicated by two items: Ins_M (“Had difficulty falling asleep?”) and Ins_E (“Woke too early and couldn’t get back to sleep?”). Cross-sectional associations at each wave and autoregressive paths across waves are presented. Cross-lagged associations are adjusted for relevant covariates: The path from adolescent loneliness (YH1) to sleep disturbances in early adulthood (H3) is adjusted for sex, age, educational plans, living alone, and mental distress. The path from loneliness at H3 to sleep disturbances at H4 is adjusted for sex, age, anxiety, and depression.^*^p < .05, ^**^p < .001. Arrows without asterisks represent non-significant paths.

Cross-lagged analyses examined the directional links between loneliness and sleep disturbances over time. In the adjusted model, loneliness at YH1 did not predict sleep disturbances at H3 (β = -0.041, *p*=.416) after adjusting for sex, age, educational plans, living status, anxiety, and depression, nor did loneliness at H3 predict sleep problems at H4 (β = -0.159, *p*=.389) after adjusting for sex, age, anxiety and depression. In contrast, sleep disturbances at YH1 did not predict at H3 (β = 0.060, *p*=.282), but sleep disturbances at H3 significantly predicted loneliness at H4 (β = 0.151, *p*=.026), even after adjusting for sex, age, anxiety, and depression (Figure 3). Most directional associations between loneliness and sleep disturbances became non-significant after adjusting for anxiety and depression, showing that anxiety and depression explain the link between loneliness and sleep disturbances.

### Cross-sectional associations between loneliness and sleep disturbance at YH1, H3, and H4

Figure 2 presents cross-sectional associations between loneliness and sleep disturbances at YH1, H3, and H4. Loneliness at YH1 was significantly associated with sleep disturbances at that time point (β = 0.218, *p*=.012). No significant association was observed at H3 (β = 0.075, *p*=.43), but loneliness at H4 was significantly related to concurrent sleep disturbances (β = 0.39, *p*<.001). Sleep disturbances at YH1 significantly predicted loneliness at the same time point (β = 0.138, *p*=.026). There was no significant association at H3 (β = 0.068, *p*=.317), but sleep disturbances at H4 were significantly linked to concurrent loneliness (β = 0.201, *p*<.001) (Figure 2).

### Mediation analyses: testing the underlying mechanism via anxiety and depression

**Figure 4:**
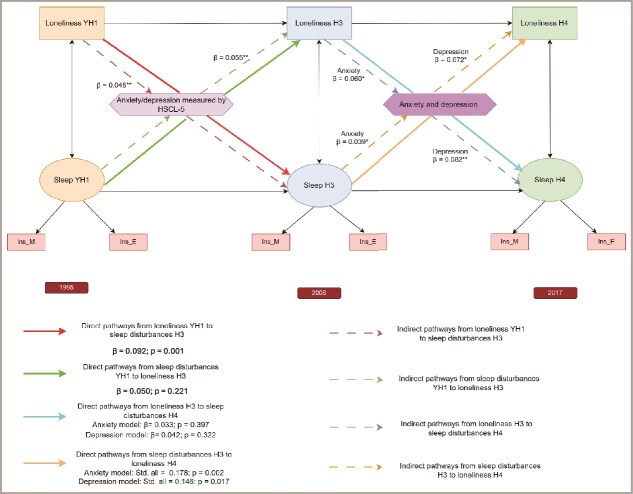
Direct and indirect pathways linking loneliness and sleep disturbances across adolescence and adulthood in the HUNT study (YH1: Young-HUNT1 in 1995, H3: HUNT3 in 2006, H4: HUNT4 in 2017). This conceptual diagram illustrates the reciprocal longitudinal associations between loneliness and sleep disturbances and the direct and indirect pathways linking loneliness and sleep disturbances across adolescence and adulthood. Anxiety and depression are positioned as central mechanisms, representing mediators of the loneliness–sleep relationship. Solid arrows represent direct pathways between loneliness and sleep disturbances with across waves. Dotted arrows represent indirect pathways operating through anxiety and depression, which were modeled as mediators of the loneliness–sleep association. Sleep disturbances were indicated by two items: Ins_M (“Had difficulty falling asleep?”) and Ins_E (“Woke too early and couldn’t get back to sleep?”). ^*^p < .05, ^*^p < .001.

The mediation analyses support a mediated pathway linking loneliness and sleep disturbances through anxiety and depression, in both directions. Table 2 shows the indirect and direct pathways linking loneliness and sleep disturbances across life stages through anxiety and depression. We found that loneliness at YH1 was associated with sleep disturbances at H3 both directly (β = 0.092, *p*=.001) and indirectly through anxiety and depression (β = 0.048, *p*<.001), with a total effect of β = 0.140 (*p*<.001). Similarly, sleep disturbances at YH1 indirectly predicted loneliness in H3 through anxiety and depression (β = 0.055, *p*=.030), with a non-significant direct effect (β = 0.050, *p*=.178) and a total effect of β = 0.105 (*p*=.001). In later adulthood, loneliness at H3 affected sleep disturbances at H4 mainly through anxiety (indirect effect β = 0.060, *p*=.001) and depression (β = 0.082, *p*<.001), with no significant direct effects. Conversely, sleep disturbances at H3 significantly predict loneliness at H4 both directly (β = 0.178, *p*=.002 in the anxiety model; β = 0.146, *p*=.017 in the depression model) and indirectly through higher anxiety (β = 0.039, *p*=.042) and depression (β = 0.072, *p*=.004) (Figure 4). The mediation analyses showed that anxiety and depression accounted for approximately one-third (33%) of the association between loneliness at YH1 and sleep disturbances at H3, indicating partial mediation. In the reverse direction, more than half (55%) of the effect of sleep disturbances at YH1 on loneliness at H3 was mediated through anxiety and depression, suggesting strong mediation. In adulthood, the effects of loneliness at H3 on subsequent sleep disturbances at H4 were almost entirely explained by anxiety and depression, which mediated about 65% and 66% of the total effects, respectively. Conversely, the associations from sleep disturbances at H3 to loneliness at H4 were partially mediated by anxiety (18%) and depression (33%). Detailed estimates of the values of the proportion mediated for each pathway, are presented in Supplementary Table S4. Taken together, these results highlight that anxiety and depression are key mechanisms linking loneliness and sleep disturbances across life stages. All mediation models showed satisfactory fit to the data (CFI and TLI ≥ 0.85, RMSEA ≤0.07, SRMR ≤0.05), indicating that the specified pathways adequately represented the observed associations.

**Table 2:** Indirect and direct pathways linking loneliness and sleep disturbances across life stages through anxiety and depression

Predictor → Outcome	Pathway Type	Mediator(s)	Standardized Effect (β or Std.all)	*p*-value
Loneliness → Sleep Disturbances (*Life stage: Adolescence to early adulthood (YH1-H3))*				
	Direct	—	0.092	.001
	Indirect	Anxiety and depression (HSCL-5)	0.048	< .001
	Total	—	0.140	< .001
Sleep Disturbances → Loneliness (*Life stage: Adolescence to early adulthood (YH1-H3))*				
	Direct	—	0.050	0.219
	Indirect	Anxiety and depression (HSCL-5)	0.055	< .001
	Total	—	0.105	.001
Loneliness → Sleep Disturbances (*Life stage: Early adulthood to mid-adulthood (H3-H4))*				
	Direct (Anxiety model)	—	0.033	0.397
	Direct (Depression model)	—	0.042	0.322
	Indirect	Anxiety	0.060	.001
	Indirect	Depression	0.082	< .001
Sleep Disturbances → Loneliness (*Life stage: Early adulthood to mid-adulthood (H3-H4))*				
	Direct (Anxiety model)	—	0.178	.002
	Direct (Depression model)	—	0.146	.017
	Indirect	Anxiety	0.039	.042
	Indirect	Depression	0.072	.004

All effects are standardized. During adolescence, links between loneliness and sleep were largely indirect via anxiety and depression measured by HSCL-5. In adulthood, anxiety and depression both mediated these associations, with sleep disturbances showing additional direct effects on later loneliness. HSCL-5: 5-Item Hopkin Symptom Checklist.

YH1: Young-HUNT1 (adolescence), H3: HUNT3 (early adulthood), H4: HUNT4 (mid-adulthood)

### Sensitivity analyses

On including the social-context variables (number of friends and sibling relationship quality) in the sensitivity analyses, we found some changes in the beta values for all models included. However, the nature of associations remained the same. (Results not shown).

### Summary of results

Both loneliness and sleep disturbances showed substantial stability across adolescence and adulthood, indicating that individuals who were lonely or experienced sleep disturbances at one time point tended to report similar difficulties later in life. Cross-sectional analyses showed that loneliness and sleep disturbances were significantly associated within the same wave during adolescence and later adulthood, but not in early adulthood. Cross-lagged analyses revealed limited evidence that loneliness predicted subsequent sleep disturbances; however, sleep disturbances in early adulthood consistently predicted higher loneliness in later adulthood, even after accounting for demographic and psychological factors. The associations between loneliness and sleep disturbances became non-significant after adjusting for anxiety and depression, suggesting that much of the association between earlier loneliness and later sleep and vice versa may be explained by shared psychological processes, specifically anxiety and depression, rather than a direct effect. Therefore, further mediation analyses were performed which showed that anxiety and depression both partially and strongly accounted for the longitudinal links between loneliness and sleep disturbances. Together, these findings suggest that loneliness and sleep disturbances are interconnected across life stages, with mental health symptoms playing a key role in their reciprocal relationship.

## Discussion

In this longitudinal study spanning across two decades, we examined the temporal dynamics and reciprocal associations between loneliness and sleep disturbances from adolescence into early and mid-adulthood. Our findings reveal within-construct stability in both loneliness and sleep disturbances across time. While cross-sectional associations between these constructs were evident in adolescence and mid-adulthood, we observed no consistent longitudinal (cross-lagged) pathways from loneliness to sleep disturbances. However, sleep disturbances in early adulthood significantly predicted increased loneliness in mid-adulthood, after adjusting for relevant covariates. Further analyses suggested that anxiety and depression mediated these longitudinal associations, indicating their role as underlying mechanisms. The sensitivity analyses (not shown in results) done to examine conceptual overlap between social-context variables and loneliness suggest that these variables capture meaningful social-context variance not fully explained by the loneliness construct itself. Therefore, we retained them in the final models to preserve both conceptual and statistical integrity.

### Cross-sectional associations between loneliness and sleep disturbances

Consistent with previous cross-sectional studies [8, 19, 22, 82], we found significant cross-sectional associations between loneliness and sleep disturbances in adolescence and mid-adulthood. However, unlike earlier findings [83], these associations were absent in early adulthood (H3), suggesting possible fluctuations in the strength of this relationship across the life course. This unexpected attenuation in early adulthood warrants further exploration. One explanation is that early adulthood involves major transitions like leaving home, pursuing education, forming new social circles, or starting work, that may buffer the psychological effects of loneliness [1]. Social roles in this phase are often fluid, and individuals may be more resilient to transient loneliness, especially when accompanied by optimism or agency. Alternatively, sleep disturbances in early adulthood may be more influenced by lifestyle factors like irregular sleep patterns, substance use, or academic/work stress, which are less directly linked to emotional well-being [25]. These findings underscore the importance of considering developmental timing and context. Methodological factors may also explain the finding, which is limitations in adjusting for confounders at H3 due to missing or inconsistently measured data that may have introduced unmeasured confounding [84]. Future research should use harmonized data and methods to handle attrition and missingness to clarify life-course patterns in the loneliness–sleep association.

### Stability and change in loneliness and sleep disturbances

Our model revealed moderate to high temporal stability in both loneliness and sleep disturbances. Loneliness persisted from adolescence to early adulthood and into mid-adulthood, though stability declined slightly over time. This aligns with research suggesting that loneliness, while situational for some, can become trait-like when rooted in early life [8]. Loneliness experienced during adolescence may foster prolonged maladaptive social cognitions about both self and others, leading to persistent loneliness even when social conditions improve or even in the presence of social relationships [8, 31]. Over time, these individuals may become more prone to interpret social cues negatively and withdraw socially, reinforcing a cycle of loneliness that becomes less dependent on immediate context and more embedded in personality [31], explaining the moderate-to-strong stability in loneliness in this study sample.

Sleep disturbances, on the other hand, demonstrated notable stability over time, with particularly strong continuity between early and mid-adulthood, and moderate stability from adolescence to early adulthood. This pattern aligns with developmental and clinical perspectives on sleep. During adolescence, the brain undergoes significant developmental changes, particularly in regions involved in emotional regulation and sleep–wake cycles [85]. Key areas include the prefrontal cortex, which continues to develop in ways that enhance executive functioning and emotional regulation [86, 87]; maturation and increased activity of the subcortical regions such as the amygdala, hippocampus, and striatum, which modulate emotional reactivity and reward processing [88]; and structural changes in the hypothalamus, particularly the suprachiasmatic nucleus (SCN), which serves as the brain’s master circadian clock and regulates sleep–wake rhythms [89]. At the same time, psychosocial transitions, such as increased academic demands, changing peer relationships, and shifts in family dynamics, can create variability in both emotional well-being and sleep patterns [90, 91]. These biological and social changes can lead to instability in sleep patterns over time, with some adolescents developing chronic sleep disturbances, while others experience temporary or situational issues that resolve as they mature [90, 92]. In early adulthood, however, sleep patterns become more ingrained, and disturbances may turn chronic, especially without intervention [93, 94]. This chronicity is often reinforced by comorbid anxiety, depression, or loneliness [19, 95], intensification with age [96, 97], as well as behavioral and physiological mechanisms such as poor sleep habits and hygiene, HPA axis dysregulation, and increased allostatic load [98–100]. Cognitive factors like rumination and negative sleep hygiene and beliefs may also maintain arousal, contributing to persistent problems [101].

### Cross-lagged pathways

Our findings build on prior research showing that loneliness predicts sleep disturbances over short- to medium-term periods [34, 35, 82, 102], but suggest that this link may not extend across longer spans like adolescence to mid-adulthood. One possible explanation lies in the contextual and dynamic nature of loneliness, i.e. it may fluctuate in response to life events, social roles, and environmental factors, especially during adolescence and early adulthood, which are periods of major social transitions [103–105]. In this context, early loneliness may not capture a long-term vulnerability, but rather a temporary state that becomes less predictive as individuals’ social circumstances evolve. Additionally, mechanisms like hypervigilance and stress reactivity may also weaken over time or be counterbalanced by protective factors such as social support or coping strategies, or become overshadowed by other determinants of sleep, such as work stress, parenting demands, or health changes [105–108]. Moreover, limitations of our data such as single-item measures, one-time assessments, and missing covariate data, particularly in early adulthood (H3), may have reduced our ability to detect long-term effects [84]. Future studies using repeated, multi-item measures are needed to assess whether persistent loneliness poses sustained risks for sleep.

Conversely, the reverse association of sleep disturbances predicting loneliness was significant from early to mid-adulthood, consistent with prior work [19, 22]. Poor sleep can impair emotional regulation, empathy, and sensitivity to social cues [50, 109]. Sleep-deprived individuals have been shown to be physically tired, misread social cues, respond more negatively in social interactions, and exhibit reduced prosocial behavior, all of which may impair the development or maintenance of meaningful relationships. Over time, such impairments may foster social withdrawal, reduced perceived social support, or even interpersonal conflict, which can, in turn, increase the risk of experiencing loneliness [110]. Our findings also showed that most cross-lagged directional associations between loneliness and sleep disturbances became non-significant after adjusting for anxiety and depression, suggesting that these affective symptoms may explain the underlying mechanisms of their longitudinal association.

### Mediation analyses

Our mediation analyses highlight key psychological pathways linking loneliness and sleep disturbances across the life course. Loneliness and sleep problems each showed lasting, reciprocal effects, primarily through symptoms of anxiety and depression. From adolescence to early adulthood, loneliness influenced later sleep both directly and indirectly through anxiety and depression, while sleep disturbances also contributed to later loneliness through similar pathways. From early to mid-adulthood, loneliness was linked to later sleep mainly through its association with anxiety and depression, whereas sleep disturbances predicted later loneliness both directly and indirectly. These findings support models that position internalizing symptoms as central mediators in the interplay between loneliness and sleep. Loneliness may increase vulnerability to anxiety and depression by fostering worry, rumination, and hopelessness, i.e. processes that, in turn, disrupt sleep [47, 49, 54]. Conversely, sleep problems can impair emotional regulation and reduce social engagement, thereby reinforcing loneliness through internalizing symptoms [50]. The significant indirect effects and good model fit indices further support this psychologically mediated, bidirectional framework.

From a public health standpoint, our findings emphasize the importance of early identification and intervention to prevent the development of maladaptive trajectories and the cascading social and emotional consequences of chronic sleep and loneliness. Moreover, targeting anxiety and depressive symptoms as part of early preventive strategies could help disrupt this reinforcing cycle, promoting better long-term mental and physical health outcomes.

### Strengths and limitations

This study leverages the extensive, population-based HUNT cohort, enabling the examination of longitudinal associations between sleep disturbances and loneliness across critical developmental stages, i.e. from adolescence through early and into mid-adulthood. The use of SEM with latent variables enhances the precision of our estimates by accounting for measurement errors and allowing for the simultaneous assessment of stability and directional pathways. Notably, the identification of a significant pathway from early adult sleep disturbances to increased loneliness in mid-adulthood provides novel insight into the potential long-term social consequences of sleep problems, highlighting a directionality that has been underexplored in prior research. While prior research has identified plausible mechanisms linking sleep disturbances to social difficulties, our study provides novel evidence that these effects may emerge over longer developmental timescales. The predictive association from early adult sleep problems to loneliness a decade later underscores the potential for delayed and cumulative social consequences of untreated sleep difficulties, which is an underexplored area. Moreover, the directional asymmetry observed in our findings, whereby sleep predicts later loneliness, but not the reverse, adds conceptual nuance to the sleep–loneliness literature and highlights sleep as a potential upstream intervention target for social well-being.

Despite these strengths, several limitations should be acknowledged. First, loneliness was assessed using a single-item measure at each time point, which limits the ability to capture its multidimensional nature and may have attenuated associations due to measurement error and violation of invariance. Similarly, the sleep disturbances construct was derived from only two items, focusing on the difficulty of initiating and maintaining sleep, while excluding other relevant aspects such as nocturnal awakenings, duration, quality, and daytime consequences resulting in information bias. Although these items showed a good model fit, the restricted measurement scope limits construct validity.

Second, attrition across waves introduces potential selection bias. While the participation rate in YH1 was high, the overall participation in H3 was lower and thus only 31.5% of individuals aged 20–29 participated in H3, largely due to migration from the county for education and ineligibility for invitation. Moreover, individuals with chronic diseases, poor self-rated health, or smoking habits were more likely to drop out between H3 and H4, which could underestimate associations if these factors are related to sleep or loneliness. These patterns suggest that attrition is not random but reflects a consistent loss of more vulnerable individuals across time points, socially, psychologically, and physically, leading to an analytic sample that is more socioeconomically stable and psychologically resilient than the original cohort. Although SEM techniques allow for the inclusion of participants with partially missing data, the selective attrition may still influence the generalizability of our findings.

Third, several relevant covariates, including number of children, relationship status, living status, and socioeconomic status, were not consistently available across all time points, particularly in H3. This may result in residual confounding, especially in the modeling of bidirectional associations. One consideration regarding the generalizability of our findings is the inherently relatively low levels of loneliness, anxiety, depression, and sleep disturbances observed in the sample (Table 1). This pattern is consistent with the broader HUNT dataset and reflects characteristics of the Norwegian population from which the sample is drawn (Young HUNT 1, HUNT 3, and HUNT 4). While this is not a limitation per se, it may influence the applicability of the results to populations with higher prevalence of these conditions. Finally, as with all observational studies, causality cannot be inferred, and unmeasured variables may have influenced the reported associations.

## Conclusions

In summary, our study highlights the enduring nature of both loneliness and sleep disturbances from adolescence to adulthood. While these constructs are closely linked cross-sectionally, particularly in adolescence and mid-adulthood, we found limited evidence that adolescent loneliness predicts future sleep problems. In contrast, early adult sleep disturbances are significantly associated with loneliness in midlife, underscoring the potential of sleep as a modifiable risk factor for social disconnection later in life. Importantly, our findings suggest that symptoms of anxiety and depression mediate these associations, indicating that they are key mechanisms linking loneliness and sleep disturbances over time. Future studies should explore the underlying role of biological mechanisms and psychological pathways (e.g. stress physiology, circadian disruption, emotional regulation) in the longitudinal association between loneliness and sleep. Investigating whether these pathways differ across different subgroups defined by sex, SES, physical activity or resilience levels could help identify adolescents most at risk. Finally, extending follow-up into later adulthood would provide insight into the enduring impact of adolescent loneliness and sleep disturbances on broader life outcomes and overall well-being.

### Clinical and public health implications

These findings have notable implications for prevention and intervention efforts. The strong temporal stability observed suggests that early interventions targeting loneliness and sleep disturbances, particularly insomnia symptoms of difficulty falling asleep and early morning awakening and especially during adolescence and early adulthood, may prevent the chronicity of these problems. Moreover, our results highlight sleep disturbances as an independent driver of later loneliness, supporting calls for integrated treatment approaches that address both sleep and psychosocial well-being. Evidence-based interventions, such as Cognitive Behavioral Therapy for Insomnia (CBT-I), sleep hygiene education, stimulus control, and mindfulness-based strategies could be adapted to address both sleep and psychosocial well-being. Integrated interventions that simultaneously target mental distress, loneliness, and sleep disturbances may be particularly effective in promoting long-term mental health. From a public health perspective, our results emphasize the need for holistic mental health strategies that consider both affective and somatic symptoms, supporting social functioning and overall well-being across the life course.

## Supplementary Material

Supplementary_file_accepted_zpag004

## Data Availability

The HUNT databank provided the data used in this study, but access is restricted. Data can be accessed by approaching the corresponding author, Nayan Parlikar (nayan.d.parlikar@ntnu.no) and obtaining permission from HUNT, the Regional Ethical Committee, and the Norwegian Data Protection Authority. All the subjects of this study are stored in HUNT data using a personal identification number as an ID number. The HUNT Research Centre stores and uses these data with authorization from the Norwegian Data Inspectorate without breaking participant privacy. The researcher will always receive an anonymous dataset after approval from the Regional Ethical Committee and HUNT Research Centre. For more information about HUNT data see https://www.ntnu.edu/hunt/data.
