# Supplementary material for: Reciprocal effects between loneliness and sleep disturbances from adolescence to mid-adulthood: the HUNT study
Source: Sleep Adv. 2026 Jan 12;7(1):zpag004. doi: 10.1093/sleepadvances/zpag004 (PMC12936869; doi:10.1093/sleepadvances/zpag004)
Supplement: Supplementary_file_accepted_zpag004 [file supplementary_file_accepted_zpag004.docx]

**Reciprocal Effects Between Loneliness and Sleep Disturbances from Adolescence to Mid-Adulthood: The HUNT Study**

Nayan Parlikar^1^, Joanna McHugh Power^5^, Philip Hyland^5^, Andrew N. Coogan^5^, Kirsti Kvaløy^1,2,3^, Linn Beate Strand^1^, Geir Arild Espnes^1^, Steinar Krokstad^1,2,3,4^, Unni Karin Moksnes^1^

**Corresponding author:** Nayan Parlikar

Address: Kolstadflata 37C, H0301

Trondheim 7098, Norway

([parlikarnayan@gmail.com](mailto:parlikarnayan@gmail.com); nayan.d.parlikar@ntnu.no)

^1^Department of Public Health and Nursing, Faculty of Medicine and Health Sciences, Norwegian University of Science and Technology, Trondheim, Norway

^2^HUNT Research Centre, Department of Public Health and Nursing, Norwegian University of Science and Technology, Levanger, Norway

^3^Levanger Hospital, Nord-Trøndelag Hospital Trust, Levanger, Norway

^4^Clinic for Mental Health and Substance Use, Nord-Trøndelag Hospital Trust

^5^Department of Psychology, Maynooth University, Kildare, Ireland

Supplementary information

1. **Attrition and Selection bias**

Attrition over two decades was more pronounced among individuals with early indicators of psychological distress, poor social relationships, and unhealthy lifestyle factors. Between Young-HUNT1 and HUNT3, participants who dropped out were more likely to be male, from lower socioeconomic backgrounds (proxied by low educational attainment plans), not living with parents or family, and from divorced households. They also reported lower life and school satisfaction, fewer close friendships, poorer self-rated health, limited outdoor activity, and lower participation in organized social groups **(Table S1)**. Between HUNT3 and HUNT4, dropout was associated with financial strain, workplace problems, high BMI, and high anxiety and depression symptoms **(Table S2)**. This selective loss likely biases the analytic sample toward individuals with greater psychological resilience and more stable life circumstances, leading to conservative estimates of the associations examined. The true long-term impact of loneliness on adult mental and physical health may therefore be stronger than observed.

**Table S1:** **Participant Attrition Between Young HUNT 1 and HUNT3**

This table presents the attrition rates between the Young HUNT 1 and HUNT3 waves. It includes the number and percentage of participants who were lost to follow-up, along with demographic and baseline characteristics of those who remained versus those who dropped out. This information helps assess potential biases due to differential attrition across the study period.

**Table S1:** Participant Attrition Between Young HUNT 1 and HUNT3 Waves

| **Baseline variable (Young-HUNT1)** | **Retained at HUNT3 (N= 3358)** | **Lost to follow-up (N=5622)** | **p-value** |
| --- | --- | --- | --- |
| Sex (N,% Male) | 1439 (42.9) | 3078 (54.7) | <0.001 |
| SES (Low education plans) | 1887 (56.2) | 3328 (59.2) | <0.001 |
| Not living with parents | 176 (5.2) | 456 (8.1) | <0.001 |
| Parent’s civil status (Divorced) | 531 (15.8) | 1210 (21.5) | <0.001 |
| Self-rated health (Poor) | 305 (9.1) | 663 (11.8) | <0.001 |
| Life satisfaction (Poor) | 564 (16.8) | 1096 (19.5) | <0.001 |
| Number of friends (None) | 34 (1) | 109 (2) | <0.001 |
| Low physical activity | 532 (15.8) | 997 (17.7) | <0.014 |
| Low social participation | 42 (1.3) | 106 (1.9) | <0.021 |
| Low school satisfaction | 1242 (37) | 2484 (44.2) | <0.001 |

SES: Socioeconomic status proxied by educational plans among adolescents at Young-HUNT1

**Table S2: Participant Attrition Between HUNT3 and HUNT4**

This table provides similar information for the transition from HUNT3 to HUNT4. It outlines the number and proportion of participants who continued in the study versus those who dropped out and compares characteristics across these groups. These comparisons help evaluate the representativeness of the longitudinal sample across later waves.

**Table S2:** Participant Attrition Between HUNT3 and HUNT4

| **Baseline variable (HUNT3)** | **Retained at HUNT4 (N=2192)**  **N (%)** | **Lost to follow-up (N=1166)**  **N (%)** | **p-value** |
| --- | --- | --- | --- |
| Economic problems | 206 (23)  (N= 895) | 96 (31.4)  (N= 305) | 0.002 |
| Workplace problems | 240 (27)  (N= 892) | 105 (34.3)  (N=306) | 0.046 |
| Anxiety | 140 (15.6)  (N=898) | 66 (21.7)  (N=304) | 0.014 |
| Depression | 34 (4.7)  (N=719) | 27 (11.2)  (N=241) | <0.001 |
| High BMI (≥ 25) | 616 (46.2)  (N=1332) | 308 (54.5)  (N=565) | <0.001 |

BMI: Body mass index

Anxiety symptoms at HUNT3 were assessed using the Hospital Anxiety and Depression Scale: Anxiety subscale (HADS-A), with a score of ≥8 indicating symptoms of anxiety. Depressive symptoms were measured using the Depression subscale (HADS-D) of the same instrument, with a score of ≥8 indicating symptoms of depression. These thresholds are commonly used to identify individuals with elevated symptom levels in population-based studies (1, 2).

1. **Fit indices for different models**

**Table S3: Fit indices for measurement, autoregressive, and cross-lagged structural equation models examining associations between loneliness and sleep disturbances across three waves**

This table presents model fit statistics (χ², CFI, TLI, RMSEA, and SRMR) for each step of the longitudinal modeling process. The measurement model assessed the latent structure and item loadings for sleep disturbances at each wave. The autoregressive model incorporated stability paths of loneliness and sleep disturbances across waves, while the final cross-lagged model included reciprocal associations between loneliness and sleep disturbances, adjusted for relevant covariates at each time point.

**Table S3:** Fit indices for measurement, autoregressive, and cross-lagged structural equation models examining associations between loneliness and sleep disturbances across three waves

| Model | χ² (df) | CFI | TLI | RMSEA (90% CI) | SRMR |
| --- | --- | --- | --- | --- | --- |
| Sleep disturbances as a latent variable | 24.52 (15) | 0.982 | 0.912 | 0.064 (0.042, 0.089) | 0.019 |
| Baseline autoregressive model (loneliness & sleep disturbances) | 165.36 (77) | 0.943 | 0.915 | 0.036 (0.029, 0.044) | 0.038 |
| Cross-lagged model: loneliness to sleep disturbances | 71.23 (33) | 0.947 | 0.912 | 0.036 (0.025, 0.048) | 0.028 |
| Cross-lagged model:  sleep disturbances to loneliness | 64.17 (39) | 0.970 | 0.951 | 0.027 (0.014, 0.039) | 0.027 |
| Cross-sectional: loneliness to sleep disturbances | 131.48 (81) | 0.944 | 0.923 | 0.029 (0.019–0.037) | 0.028 |
| Cross-sectional: Sleep disturbances to loneliness | 326.59 (134) | 0.895 | \| 0.862 \| \| --- \| | 0.043 (0.038–0.049) | 0.051 |

1. **Detailed estimates of the direct, indirect, and total effects, along with the proportion mediated for each pathway**

**Table S4: Proportion of the total effect mediated by anxiety, and depression in the associations between loneliness and sleep disturbances across life stages**

This table presents detailed estimates of the direct, indirect, and total effects, along with the proportion mediated for each pathway. The purpose of this table is to provide a comprehensive breakdown of the mediation analysis, enabling readers to evaluate the relative contribution of each pathway to the overall effect from loneliness to sleep disturbances and vice versa and to assess the extent to which the observed associations are mediated through anxiety and depression.

**Table S4:** Proportion of the total effect mediated by anxiety, and depression in the associations between loneliness and sleep disturbances across life stages

| Life Stage | Predictor → Outcome | Mediator(s) | Direct Effect (β) | Indirect Effect (β) | Total Effect (β) | Proportion Mediated  (% = Indirect ÷ Total × 100) |
| --- | --- | --- | --- | --- | --- | --- |
| Adolescence → Adulthood (YH1–H3) | Loneliness → Sleep Disturbances | Anxiety and depression (HSCL-5) | 0.092 | 0.048 | 0.140 | **34.29%** |
|  | Sleep Disturbances → Loneliness | Anxiety and depression (HSCL-5) | 0.050 | 0.055 | 0.105 | **52.38%** |
| Adulthood (H3–H4) | Loneliness → Sleep Disturbances | Anxiety | 0.033 | 0.060 | 0.093 (≈ 0.033 + 0.060) | **64.5%** |
|  | Loneliness → Sleep Disturbances | Depression | 0.042 | 0.082 | 0.124 (≈ 0.042 + 0.082) | **66.1%** |
|  | Sleep Disturbances → Loneliness | Anxiety | 0.178 | 0.039 | 0.217 | **18.0%** |
|  | Sleep Disturbances → Loneliness | Depression | 0.146 | 0.072 | 0.218 | **33.0%** |

HSCL-5: 5-Item Hopkin Symptom Checklist. Anxiety and depression measured at adolescent life stage (Young-HUNT1) by HSCL-5.

YH1–H3 = longitudinal association from adolescence (Young-HUNT1) to early adulthood (HUNT3); H3–H4 = longitudinal association from early to mid-adulthood (HUNT3 to HUNT4). Standardized direct, indirect, and total effects are presented for each pathway, along with the proportion mediated (indirect ÷ total × 100). The proportion mediated indicates the percentage of the total effect of loneliness on sleep disturbances (and vice versa) through anxiety and depression.

**References**

1. Bjelland I, Dahl AA, Haug TT, Neckelmann D. The validity of the Hospital Anxiety and Depression Scale. An updated literature review. J Psychosom Res. 2002;52(2):69–77. doi:10.1016/s0022-3999(01)00296-3

2. Zigmond AS, Snaith RP. The hospital anxiety and depression scale. Acta Psychiatr Scand. 1983;67(6):361–70. doi:10.1111/j.1600-0447.1983.tb09716.x
